# MicroRNA-133b/EGFR axis regulates esophageal squamous cell carcinoma metastases by suppressing anoikis resistance and anchorage-independent growth

**DOI:** 10.1186/s12935-018-0684-y

**Published:** 2018-11-22

**Authors:** Jin-Feng Zhu, Yi Liu, He Huang, Li Shan, Zhi-Gang Han, Jun-Yuan Liu, Ying-Long Li, Xiang Dong, Wei Zeng

**Affiliations:** 1 First Department of Lung Cancer Chemotherapy, Affiliated Cancer Hospital of Xinjiang Medical University, No. 789, East Suzhou Street, Urumqi, 830011 Xinjiang People’s Republic of China; 2 Department of Gastrointestinal Surgery, Affiliated Cancer Hospital of Xinjiang Medical University, Urumqi, 830011 People’s Republic of China; 3 Department of Cardiothoracic Surgery, Shenzhen University General Hospital, Shenzhen, 518055 People’s Republic of China; 4 Department of Histology and Embryology, Xiangya School of Medicine, Central South University, Changsha, 410013 People’s Republic of China; 5 Department of Histology and Embryology, Xinjiang Medical University, Urumqi, 830011 People’s Republic of China; 6 Institute of Cancer Prevention and Treatment, Affiliated Cancer Hospital of Xinjiang Medical University, Urumqi, 830011 People’s Republic of China; 7 Department of Hematology and Oncology, Shenzhen University General Hospital, No.1098, Xueyuan Avenue, Shenzhen, 518055 Guangdong People’s Republic of China

**Keywords:** Esophageal squamous cell carcinoma, miR-133b, Epidermal growth factor receptor, Metastases, Anoikis, Anchorage-independent growth

## Abstract

**Background:**

Anoikis resistance has been demonstrated to facilitate distant metastases of cancers. MicroRNA-133b (miR-133b) is found to be down-regulated in various tumors, including esophageal squamous cell carcinoma (ESCC), and closely correlates with the malignant phenotype of ESCC. This study aimed to evaluate the roles of miR-133b in metastases of ESCC via regulating anoikis.

**Methods:**

The expression of miR-133b and related molecules were detected in ESCC tissues and cells. The target relationship between miR-133b and epidermal growth factor receptor (EGFR) was verified by dual luciferase reporter assay. Cell proliferation was detected by 3-(4,5-dimethylthiazol-2-yl)-2,5-diphenyltetrazolium bromide assay. Anoikis and anchorage-independent growth were assessed by anoikis assay and soft agar assay. Migration and invasion were evaluated by scratch and transwell assays. The expressions of related molecules were detected by reverse transcription-quantitative polymerase chain reaction and western blotting. The in vivo results were determined by tumor xenografts in nude mice.

**Results:**

MiR-133b level was decreased in ESCC tissues and cells, which negatively correlated with EGFR, integrin β4 (ITGB4), and phosphorylated focal adhesion kinase levels. Moreover, miR-133b down-regulated EGFR expression in ESCC cells. Overexpression of miR-133b inhibited the anoikis resistance, migration, invasion and epithelial-mesenchymal transition of ESCC cells via targeting EGFR. Finally, miR-133b overexpression suppressed tumor growth and lung metastases of ESCC in vivo. ITGB4/FAK/growth factor receptor-bound protein 2 (Grb2), protein kinase B (AKT), and extracellular signal-regulated kinase (ERK) pathways were involved in the regulatory mechanisms of miR-133b/EGFR axis in ESCC metastases in vitro and in vivo.

**Conclusions:**

The results suggested that miR-133b/EGFR axis regulated metastases of ESCC by affecting anoikis resistance via ITGB4/FAK/Grb2, AKT, and ERK pathways.

## Background

Esophageal squamous cell carcinoma (ESCC), as the main histological type of esophageal carcinoma, is the fourth most common cancer in China [1, 2]. ESCC has been demonstrated to have rapid progression, strongly potential invasion, high frequent metastasis and poor survival [3]. Currently, surgery, chemotherapy and radiotherapy are the common therapeutic methods for ESCC. Whereas the prognosis of ESCC is still poor and the 5-year survival rate is only about 15–25% [4, 5]. Regional or distant metastasis is a risk factor for the recurrence and poor prognosis of ESCC. Therefore, it is urgent to identify new markers and study the mechanisms for recurrence and metastasis to enhance the survival rate of ESCC patients.

MicroRNAs (miRNAs) are short endogenous non-coding RNAs that repress the expression of their target genes via binding to the 3′-untranslated regions (3′-UTRs). Growing evidences have shown that some miRNAs are aberrantly expressed and play pivotal roles in the regulation of growth and metastasis of ESCC. As one of well documented miRNAs, miR-133b has been confirmed to be a tumor suppressor that inhibits the progression of various cancers [6–8]. In ESCC, miR-133b has been also identified to be down-regulated and involved in the malignant phenotype of ESCC [9, 10]. However, the regulatory mechanisms of miR-133b in the development and progression of ESCC have not been fully elucidated.

Epidermal growth factor receptor (EGFR) that belongs to the human epidermal growth factor receptor family, acts as a proto-oncogene, and promotes cell growth and metastasis [11]. Previous studies demonstrated that miR-133b could restrain cell invasion and metastasis via targeting EGFR in a variety of cancer cells [12–14], including ovarian cancer, non-small-cell lung cancer, and colorectal cancer. However, whether miR-133b can modulate metastases in ESCC through regulating EGFR expression has not been elucidated.

Frisch and Francis discovered anoikis for the first time in 1994 [15]. Anoikis is a programmed cell death that prevents the growth of cells after they separate from the extracellular matrix (ECM) [16]. However, the disorder of gene expression helped the cancer cells to escape anoikis, which resulted in the survival of cancer cells in lymph, blood and facilitated their regional or distant metastasis [17, 18]. Thus, inducing anoikis of cancer cells may be an effective treatment for suppressing cancer metastasis. A previous study showed that *N*-acetylglucosaminyltransferase V gene promoted anoikis resistance during metastasis of cancers [19]. Hu et al. demonstrated that inducing caspase-mediated anoikis inhibited the progression of hepatocellular carcinoma [20]. Anchorage-independent growth is a feature of highly invasive tumor cells, because these cells have a better chance of survival and proliferation in the absence of extracellular matrix, then expand and invade adjacent tissues, and further give rise to metastasis [21]. Anoikis resistance is an important protective process for anchorage-independent growth [22]. It has been shown that promoting anchorage-independent growth contributed to the invasion and progression of lung cancer cells [23]. But so far, the roles of miR-133b/EGFR axis in the regulation of anoikis resistance and anchorage-independent growth of ESCC have not been reported.

It was shown that EGFR cooperated with integrin β4 (ITGB4) to promote anchorage-independent growth and metastasis of hepatocellular carcinoma [24]. ITGB4 belongs to the integrin family and has been reported to influence carcinoma progression via modulating anoikis. Focal adhesion kinase (FAK) is a protein tyrosine kinase that mediates cell adhesion, and the activation of FAK can ligate conformation of integrin to promote cell proliferation via downstream phosphatidylinositol 3-kinase (PI3K)/Protein kinase B (AKT) pathway, which finally lead to anoikis resistance [25]. Growth factor receptor-bound protein 2 (Grb2) is a member of integrin adhesive and critical for the malignant progression of tumors [26]. Previous study found that Grb2/extracellular signal-regulated kinase (ERK)/caspase-3 signaling pathway was involved in anoikis resistance in breast cancer cells [27]. Moreover, Grb2 was suggested to bind to FAK and regulate the proliferation and invasion of melanoma [28]. AKT and ERK are two important downstream signaling pathways regulated by Grb2 and then facilitate tumor progression [29, 30]. Therefore, ITGB4/FAK/Grb2, AKT and ERK pathways participate in the regulation of anoikis resistance.

In this study, we investigated the roles of miR-133b/EGFR axis in the metastases of ESCC through modulating anoikis resistance and anchorage-independent growth via ITGB4/FAK/Grb2, AKT and ERK signaling pathways. The research was performed at the clinical, cell, and animal levels, which provided evidences for miR-133b as a therapeutic target for the treatment of ESCC metastasis.

## Methods

### Patient samples

A total of 30 pairs of ESCC tissues and corresponding adjacent normal tissues were collected from patients with informed consents in the Affiliated Cancer Hospital of Xinjiang Medical University (Urumchi, Xinjiang, China). The experimental protocol was approved by the research ethics committee of the Affiliated Cancer Hospital of Xinjiang Medical University. All patients were not received chemotherapy or radiotherapy before surgery. The characteristics of ESCC patients are shown in Table 1. The samples were immediately snapped frozen in liquid nitrogen till further analysis.

**Table 1:** Characteristics of ESCC patients enrolled in the study

Clinicopathologic characteristics	Case distribution
Age (median)	37–75 (62)
Gender	
Male	18
Female	12
Grade of differentiation	
Well	10
Moderate	13
Poorly	7
Degree of tumor invasion	
Submucosa	6
Muscularis propria	11
Adventitia	13
Lymph node metastasis	
Negative	14
Positive	16

### Cell culture and transfection

Human ESCC cell lines KYSE30, KYSE150, and ECa109 (Type Culture Collection of the Chinese Academy of Sciences, Shanghai, China) were cultured in Roswell Park Memorial Institute (RPMI) 1640 supplemented with 10% fetal bovine serum (FBS). Normal human esophageal epithelial cell line Het-1A (Cobioer Biosciences, Nanjing, Jiangsu, China) was maintained in Dulbecco’s Modified Eagle Medium (DMEM) with 10% FBS. All cells were cultured at 37 °C in a humidified atmosphere containing 5% CO_2_.

KYSE150 and ECa109 cells were transfected with miR-133b agomir/antagomir (GenePharma, Shanghai, China), or shEGFR (GenePharma) using Lipofectamine^R^ RNAiMAX Transfection Reagent (Invitrogen, Carlsbad, CA, USA) according to the instructions.

### Dual luciferase reporter assay

The plasmids containing wild-type (WT) or mutated (MUT) 3′-UTR of EGFR were obtained from GeneCopoeia (Guangzhou, Guangdong, China). HEK-293T cells were seeded into 96-well plates and co-transfected with 100 ng luciferase reporter constructs (EGFR-WT or EGFR-MUT) and 100 nM miR-133b agomir or miR-133b negative control using Lipo2000 (Invitrogen). At 48 h after the transfection, luciferase activity was assessed using a Dual-Luciferase Reporter Assay System (Promega, Madison, Wisconsin, USA). The dual luciferase reporter assay was performed for three times.

### 3-(4,5-dimethylthiazol-2-yl)-2,5-diphenyltetrazolium bromide (MTT) assay

MTT assay was performed to evaluate cell proliferation. Briefly, the transfected cells were seeded into 96-well plates and incubated for 24 h. Then at the indicated time points, 5 mg/mL MTT (Sigma, Saint Louis, MO, USA) was added into each well. After incubation for 4 h, the supernatant was removed and 200 μL of dimethyl sulfoxide (DMSO, Sigma) was added to dissolve the formazan products. The results were read at 490 nm by a microplate reader (BioTek, Winsky, Vermont, USA).

### Anoikis assessment assay

KYSE150 and ECa109 cells were seeded into poly-Hema (Sigma) pre-coated plates and incubated at 37 °C for 24 h. Then, the cells were collected, washed with cold phosphate buffer solution (PBS) and subjected to flow cytometry analysis using an Annexin V/FITC Apoptosis Detection Kit (BD Pharmingen™, Franklin lake, New Jersey, USA) according to the manufacture’s instructions.

### Soft agar cloning assay for anchorage-independent growth

About 1500 cells/well were suspended in pre-warmed culture medium containing 0.25% agarose, and seeded in culture dishes that were pre-coated with 0.5% agarose in culture medium. Cells were incubated for 2–3 weeks at 37 °C under a 5% CO_2_ atmosphere. The formed colonies were counted and imaged under a light microscope (Olympus, Tokyo, Japan).

### Scratch assay

Cell migration ability was determined by scratch assay. In brief, the transfected cells (0.5 × 10^6^ cells/well) were seeded into 6-well plates. Then, a scratch was made in the confluent monolayer cells using a 10 μL pipette tip. After washing with PBS to remove the cell debris, the images were photographed by a light microscope immediately. Then the cells were maintained in serum-free medium for 24 h at 37 °C and photographed. The migration rate of cells was measured by the following formula: (W_0h_ − W_24h_)/W_0h_ × 100%.

### Transwell assay

Cell invasion ability was assessed by transwell assay. Cells in serum-free medium were seeded in the upper compartments of transwell chambers (Corning, Corning, MA, USA) that were pre-coated with Matrigel. As a chemoattractant, the bottom compartments were added with RPMI1640 containing 20% FBS. After incubation for 16 h at 37 °C, the non-invaded cells on the upper surface were erased with a cotton swab and the invaded cells on the lower surface were fixed with 4% paraformaldehyde and stained with Giemsa. The images were photographed in random fields under a light microscope (Olympus, Tokyo, Japan).

### Tumor xenografts in nude mice

Male 7-week-old BALB/c nude mice were purchased from Chinese Academy of Science (Shanghai, China) and maintained under pathogen-free condition. All animal experiments were performed in accordance with the Guide for the Care and Use of Laboratory Animals and approved by the Institutional Ethics committee of the Affiliated Cancer Hospital of Xinjiang Medical University. To evaluate the role of miR-133b in tumor formation, 5 × 10^6^ KYSE150 and ECa109 cells that infected with lentivirus expressing miR-133b agomir or miR-133b NC (GeneChem, Shanghai, China) were injected subcutaneously into the axilla of nude mice. The mice were randomly divided into four groups (n = 5 per group): KYSE150-miR-133b NC, KYSE150-miR-133b agomir, ECa109-miR-133b NC, ECa109-miR-133b agomir. The length and width of the tumors were measured every 5 days and the tumor volume was calculated according to the formula of 0.5 × length × width^2^. At 30 days after the injection, the mice were sacrificed and the tumors were collected and weighed. To determine lung metastasis, 5 × 10^6^ the above cells were injected into the nude mice via tail vein. Thirty days later, the mice were killed and the lung tissues were collected, and stored in liquid nitrogen for further tests.

### Reverse transcription-quantitative polymerase chain reaction (RT-qPCR) assay

Total RNAs were isolated from ESCC tissues and cells by Trizol (Invitrogen, USA) and reversely transcribed using the PrimerScript RT reagent Kit (TaKaRa, Osaka, Japan). Real-time PCR was performed to assess mRNA and miRNA levels using SYBR Green RT-qPCR SuperMix Kit (Thermo Fisher Scientific, Waltham, Massachusetts, USA) with specific primers (Table 2) on AB7300 thermo-recycler (Applied Biosystems, Foster City, California, USA). Glyceraldehyde-3-phosphate dehydrogenase (GAPDH) and U6 small nuclear RNA (U6 snRNA) were used as internal controls for mRNA and miRNA, respectively. The levels of miR-133b and mRNAs were calculated by the 2^−ΔΔCt^ method.

**Table 2:** Oligonucleotide primer sets for real-time PCR

Name	Sequence (5′–3′)	Length
miR-133b-F	TTTGGTCCCCTTCAACCAGCTA	22
miR-133b-R	GTGCAGGGTCCGAGGT	16
EGFR-F	CACTGCCTCATCTCTCACCATC	22
EGFR-R	GACTCACCGTAGCTCCAGAC	20
ITGB4-F	GCGACTACACTATTGGATTTGGC	23
ITGB4-R	TGTCAGGCTGATGACGTTCTTG	22
FAK-F	CATCCCTAACCATTGCG	17
FAK-R	GCCCGTTCACCTTCTTT	17
Grb2-F	AAGACGGCTTCATTCCCAAG	20
Grb2-R	CTCTCTCGGATAAGAAAGGC	20
GAPDH-F	CAGGGCTGCTTTTAACTCTGGT	22
GAPDH-R	GATTTTGGAGGGATCTCGCT	20
U6-F	CGCAAGGATGACACGCAAATTC	22
U6-R	GTGCAGGGTCCGAGGT	16

### Western blotting assay

Proteins were extracted with radio immunoprecipitation assay (RIPA) (Beyotime, Haimen, Jiangsu, China) containing 1% phenylmethanesulfonyl fluoride (PMSF) from ESCC tissues and cells followed by centrifugation at 14,000*g* at 4  °C for 10 min. The proteins in supernatant were collected and quantified by a bicinchoninic acid (BCA) Protein Assay kit (Thermo Fisher Scientific). Then 40 µg protein samples were subjected to sodium dodecyl sulphate–polyacrylamide gel electrophoresis (SDS-PAGE) and transferred onto polyvinylidene fluoride membranes (Millipore, Massachusetts, USA). Subsequently, the membranes were incubated with 5% skim milk for 1 h to block the non-specific binding and probed with primary antibodies against EGFR (1:2000, Abcam, Cambridge, UK), ITGB4 (1:1000, Abcam), p-FAK (1:1000, Abcam), FAK (1:1000, Abcam), Fibronectin (1:1000, Abcam), Vimentin (1:1000, Cell Signaling Technology, Danvers, MA, USA), N-cadherin (1:1000, Cell Signaling Technology), E-cadherin (1:1000, Cell Signaling Technology), matrix metalloproteinase 2 (MMP-2, 1:1000, Proteintech, Rosemont, Illinois, USA), MMP-9 (1:1000, Proteintech), Grb2 (1:1000, Proteintech), p-AKT_Thr308_ (1:1000, Cell Signaling Technology), p-AKT_Ser473_ (1:2000, Cell Signaling Technology), AKT (1:1000, Cell Signaling Technology), p-ERK1/2 (1:1000, Abcam), ERK1/2 (1:1000, Abcam), GAPDH (1:5000, Proteintech) at 4 °C overnight, respectively. Then, the membranes were incubated with horseradish peroxidase-conjugated goat anti-mouse or anti-rabbit (1:5000, Beyotime) secondary antibody for 1 h at room temperature and visualized using ECL reagent (Millipore).

### Statistical analysis

All experiments were performed at least for three times with one representative experiment shown. Data were expressed as mean ± standard deviation (SD). Statistical analysis was performed using Student’s *t* test (two tailed) between two groups or one-way analysis of variance (ANOVA) followed by Tukey post hoc test for multiple comparison by SPSS software version 13.0. Differences were considered statistically significant at *p* < 0.05.

## Results

### Levels of miR-133b, EGFR, ITGB4, and FAK in ESCC tissue samples

**Fig. 1:**
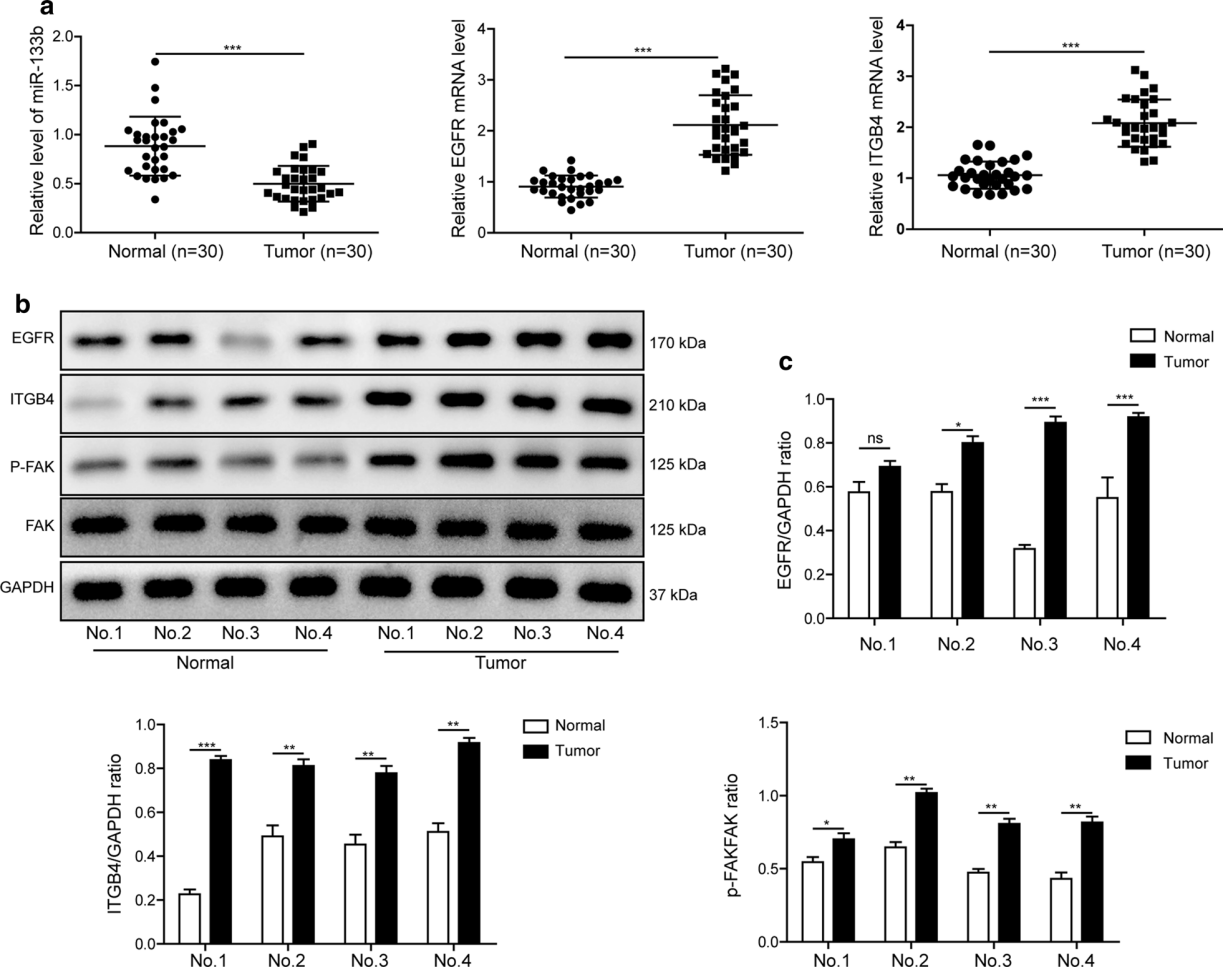
Levels of miR-133b, EGFR, ITGB4, and FAK in ESCC tissue samples. **a** The level of miR-133b, and mRNA levels of EGFR and ITGB4 in 30 pairs of ESCC tissues and corresponding adjacent normal tissues were detected by RT-qPCR. **b** The protein levels of EGFR, ITGB4, p-FAK, and FAK in ESCC tissues and adjacent normal tissues were evaluated by western blotting assay. **c** The gray-scale value of the bands were quantitatively analyzed. The experimental data were representative of three independent experiments. Results were expressed as mean ± SD. *P < 0.05, **P < 0.01 and ***P < 0.001

At first, the miR-133b level, and EGFR, ITGB4 mRNA levels in 30 pairs of ESCC tissues and adjacent normal tissues were assessed by RT-qPCR. The clinicopathological characteristics of patients with ESCC are listed in Table 1. As illustrated in Fig. 1a, compared with in normal tissues, the level of miR-133b was decreased, while EGFR and ITGB4 mRNA levels were enhanced in tumor tissues. Moreover, western blotting analysis was performed to further verify the above results. As shown in Fig. 1b, c, the protein levels of EGFR, ITGB4 and p-FAK were increased in tumor tissues, as compared with in normal tissues. From these results, we found that miR-133b level was negatively correlated with EGFR and ITGB4 levels in tumor tissues, which may participate in the metastasis of ESCC.

### Levels of miR-133b, EGFR, ITGB4, and FAK in ESCC cell lines

**Fig. 2:**
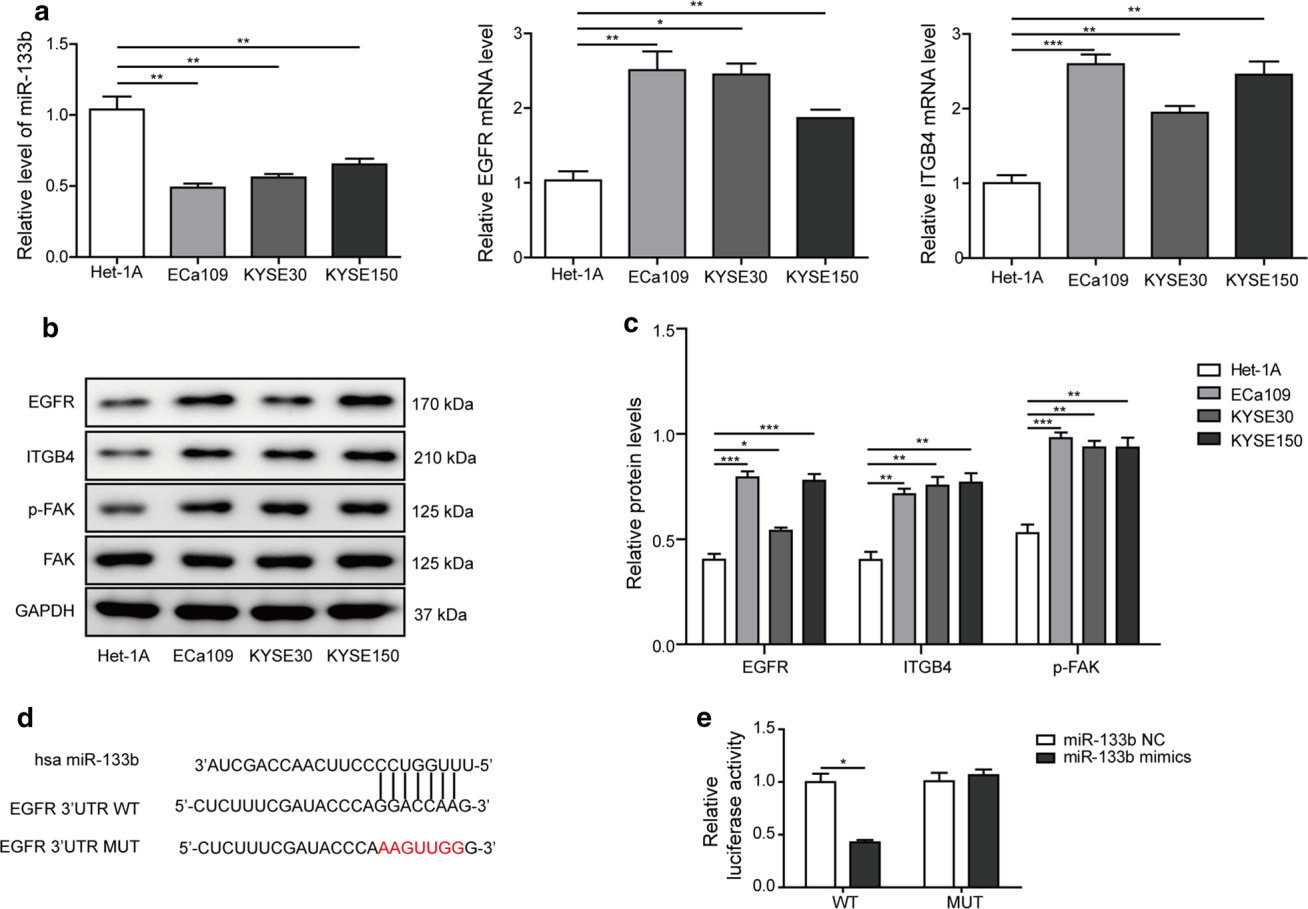
Levels of miR-133b, EGFR, ITGB4, and FAK in ESCC cell lines. **a** The level of miR-133b, and mRNA levels of EGFR and ITGB4 in ESCC cell lines and normal human esophageal epithelial cell line were detected by RT-qPCR. **b** The protein levels of EGFR, ITGB4, p-FAK, and FAK in ESCC cell lines and normal human esophageal epithelial cell line were detected by western blotting. **c** The gray-scale value of the bands were quantitatively analyzed. **d** The specific binding sites of miR-133b in 3′-UTR of EGFR mRNA, and mutation binding sites were shown. WT, wild-type; MUT, mutant type. **e** Relative luciferase activities after co-transfection with the EGFR reporter and the miR-133b agomir or miR-133b NC for 48 h. The experimental data were representative of three independent experiments. Results were expressed as mean ± SD. *P < 0.05, **P < 0.01 and ***P < 0.001

Next, we investigated the levels of miR-133b, EGFR, and ITGB4 at the cellular level in three ESCC cell lines, including KYSE150, KYSE30, and ECa109, and normal human esophageal epithelial cell line Het-1A. Consistent with the results in tissue samples, the level of miR-133b was reduced significantly, while EGFR and ITGB4 mRNA levels were strikingly increased in three ESCC cell lines, compared with in normal Het-1A cells (Fig. 2a). As expected, the protein levels of EGFR, ITGB4, and p-FAK in KYSE150, KYSE30, and ECa109 cells were distinctly enhanced (Fig. 2b, c). Thus, the negative relationship between miR-133b and EGFR, ITGB4 was further confirmed in ESCC cells. KYSE150 and ECa109 cells were selected for the following experiments.

### MiR-133b regulated EGFR expression in ESCC cell lines

**Fig. 3:**
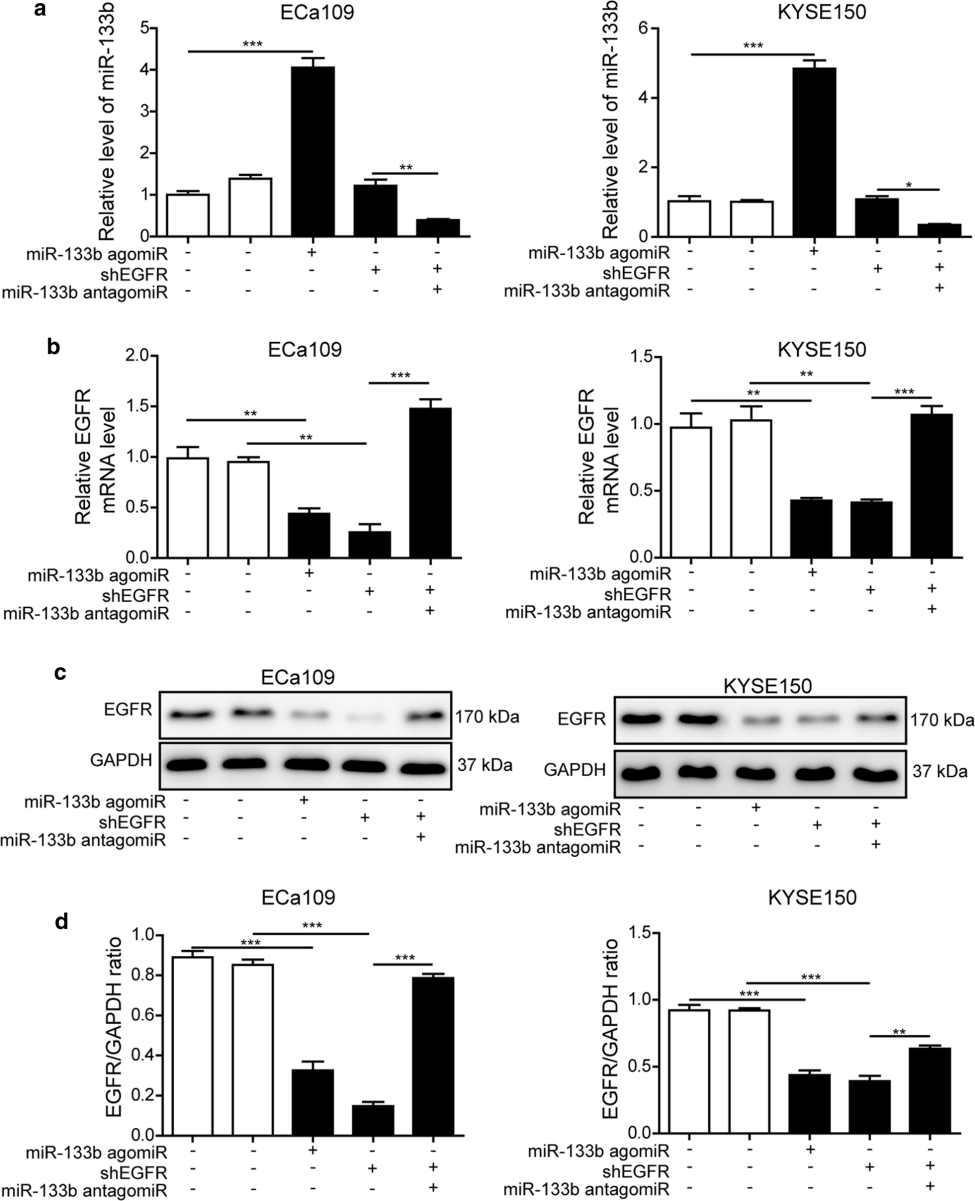
MiR-133b regulated EGFR expression in ESCC cells. **a**, **b** The level of miR-133b and mRNA level of EGFR in KYSE150 and ECa109 cells after transfection with miR-133b NC, miR-133b agomir, shEGFR, miR-133b antagomir and shEGFR were determined by RT-qPCR. **c** The protein level of EGFR in KYSE150 and ECa109 cells was detected by western blotting. **d** The gray-scale value of the bands were quantitatively analyzed. The experimental data were representative of three independent experiments. Results were expressed as mean ± SD. *P < 0.05, **P < 0.01 and ***P < 0.001

To evaluate whether EGFR is the target gene of miR-133b, dual luciferase reporter assay was performed. The WT or MUT 3′-UTR of EGFR was constructed to a luciferase system. The results showed that the luciferase activity of the WT 3′-UTR of EGFR was restrained by the overexpression of miR-133b, but that of the MUT 3′-UTR of EGFR was not affected (Fig. 2d, e). Furthermore, we investigated the regulatory role of miR-133b on EGFR expression in ESCC cells. In order to do that, agomir or antagomir of miR-133b was adopted to regulate miR-133b level and shEGFR to silence the expression of EGFR in ESCC cells. As presented in Fig. 3a, b, transfection with miR-133b agomir remarkably up-regulated cellular miR-133b level and simultaneously inhibited EGFR mRNA level in KYSE150 and ECa109 cells. Treatment with shEGFR effectively suppressed EGFR mRNA level, but had no effect on miR-133b level. However, co-transfection with shEGFR and miR-133b antagomir significantly repressed miR-133b level and reversed EGFR mRNA level induced by shEGFR (Fig. 3a, b). In addition, treatment with miR-133b agomir or shEGFR could reduce the protein level of EGFR in KYSE150 and ECa109 cells. Compared with in shEGFR group, the EGFR protein level was raised in shEGFR and miR-133b antagomir co-transfected group (Fig. 3c, d). According to these results, EGFR was verified to be the target gene of miR-133b in ESCC cells.

### MiR-133b repressed proliferation, anoikis resistance and anchorage-independent growth of ESCC cells via regulating EGFR

**Fig. 4:**
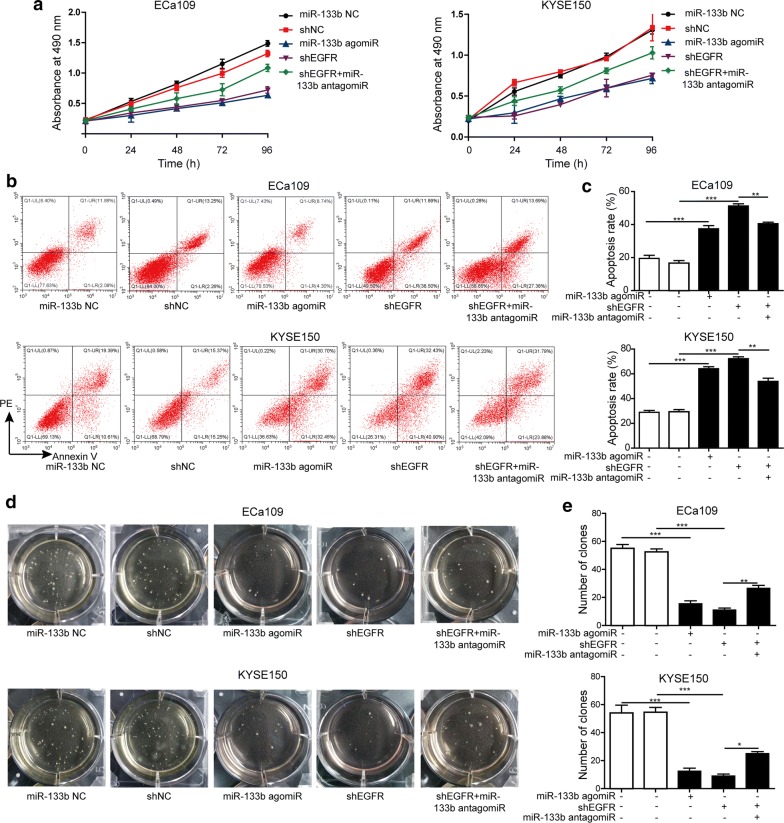
MiR-133b repressed proliferation, anoikis resistance and anchorage-independent growth of ESCC cells via regulating EGFR. **a** Proliferation of KYSE150 and ECa109 cells in different treatment groups was assessed by MTT assay. **b** Anoikis of KYSE150 and ECa109 cells that were seeded into poly-Hema pre-coated plates was detected by Annexin V/PI staining. **c** The anoikis rate of KYSE150 and ECa109 cells was calculated and shown. **d** The anchorage-independent growth in KYSE150 and ECa109 cells was assessed by soft agar cloning assay. **e** The number of clones was counted and shown. The experimental data were representative of three independent experiments. Results were expressed as mean ± SD. *P < 0.05, **P < 0.01 and ***P < 0.001

The proliferation of KYSE150 and ECa109 cells was assessed by MTT assay. It was observed that cell proliferation was restrained by miR-133b agomir or shEGFR. However, miR-133b antagomir obviously inhibited the decrease in the proliferation of KYSE150 and ECa109 cells induced by shEGFR (Fig. 4a). Moreover, miR-133b agomir or shEGFR treatment promoted apoptosis and repressed anoikis resistance of KYSE150 and ECa109 cells, whereas miR-133b antagomir weakened the effects of shEGFR treatment on anoikis resistance (Fig. 4b, c). As illustrated in Fig. 4d, e, cellular anchorage-independent growth was determined by soft agar cloning assay. The results indicated that KYSE150 and ECa109 cells that were transfected with miR-133b agomir or shEGFR formed much fewer colonies, compared with the cells in miR-133b NC or shNC group. However, miR-133b antagomir significantly increased the number of colonies that were formed by KYSE150 and ECa109 cells transfected with shEGFR. Thus, miR-133b significantly inhibited the proliferation, anoikis resistance and anchorage-independent growth of ESCC cells via regulating EGFR.

### MiR-133b suppressed migration, invasion and EMT process of ESCC cells via regulating EGFR

**Fig. 5:**
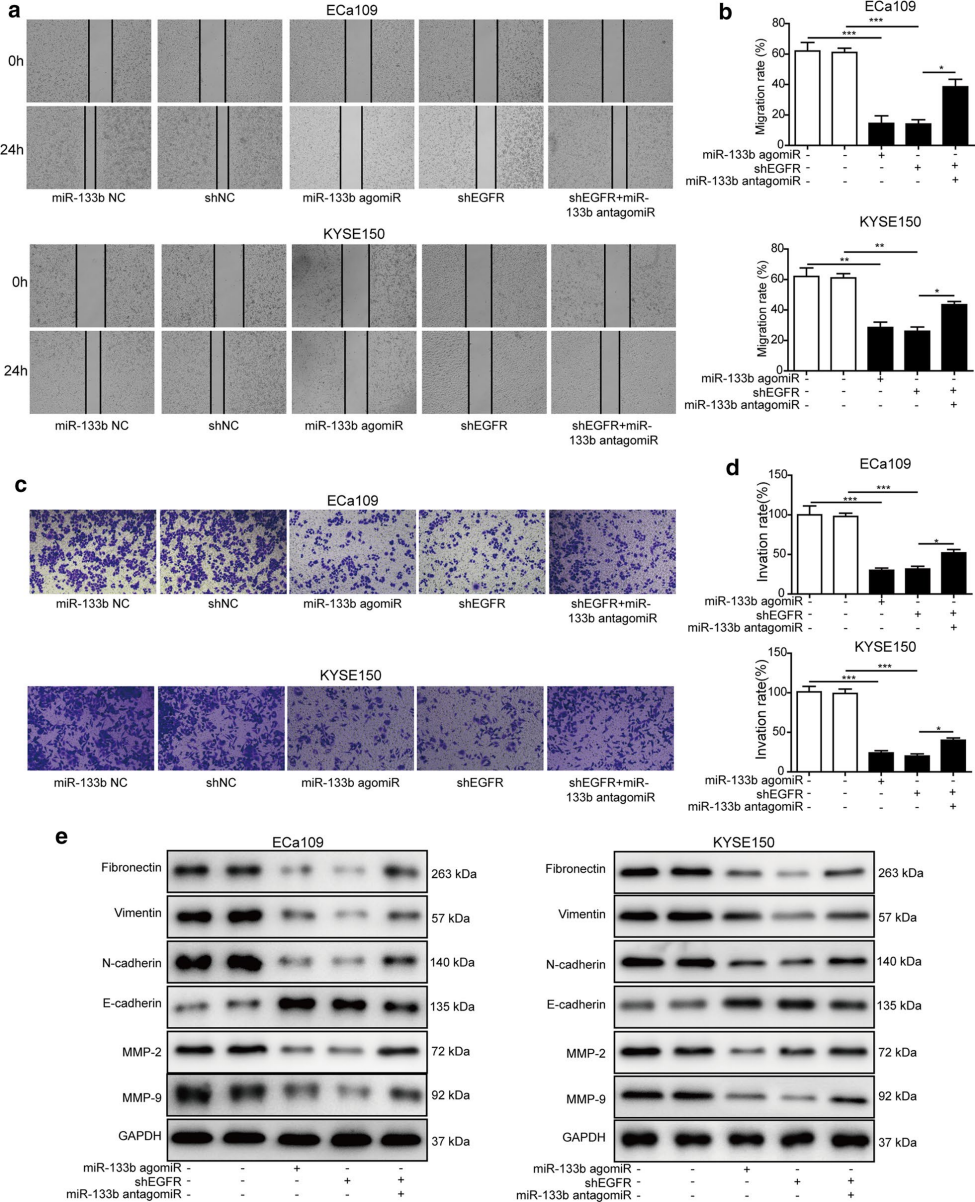
MiR-133b suppressed migration, invasion and EMT process of ESCC cells via regulating EGFR. **a** The migration ability of KYSE150 and ECa109 cells was evaluated by scratch assay. **b** The migration rate was calculated and shown. **c** The invasion ability of KYSE150 and ECa109 cells was detected by transwell assay. **d** The number of invasive cells was counted. **e** The protein levels of Fibronectin, Vimentin, N-cadherin, E-cadherin, MMP-2, and MMP-9 in KYSE150 and ECa109 cells were assessed by western blotting. The experimental data were representative of three independent experiments. Results were expressed as mean ± SD. *P < 0.05, **P < 0.01 and ***P < 0.001

The migration ability of ESCC cells was assessed by scratch assay. As presented in Fig. 5a, b, the migration ability of KYSE150 and ECa109 cells that were transfected with miR-133b agomir or shEGFR was suppressed. MiR-133b antagomir could enhance the migration ability of cells induced by shEGFR. Besides, miR-133b agomir or shEGFR evidently lessened the number of invasive KYSE150 and ECa109 cells as detected by transwell assay. But the number of invasive cells was significantly raised by miR-133b antagomir, compared with in shEGFR group (Fig. 5c, d). Moreover, the protein levels of EMT and metastasis-related markers were evaluated by western blotting analysis. As shown in Fig. 5e, the protein levels of Fibronectin, Vimentin, N-cadherin, MMP-2, and MMP-9 were decreased, while the protein level of E-cadherin was increased in KYSE150 and ECa109 cells that were treated with miR-133b agomir or shEGFR. As we expected, miR-133b antagomir could reverse shEGFR-induced the changes in these protein levels. Therefore, the migration, invasion and EMT process of ESCC cells were restrained by miR-133b via regulating EGFR.

### Regulation of miR-133b/EGFR axis in related signaling pathways of ESCC cells

**Fig. 6:**
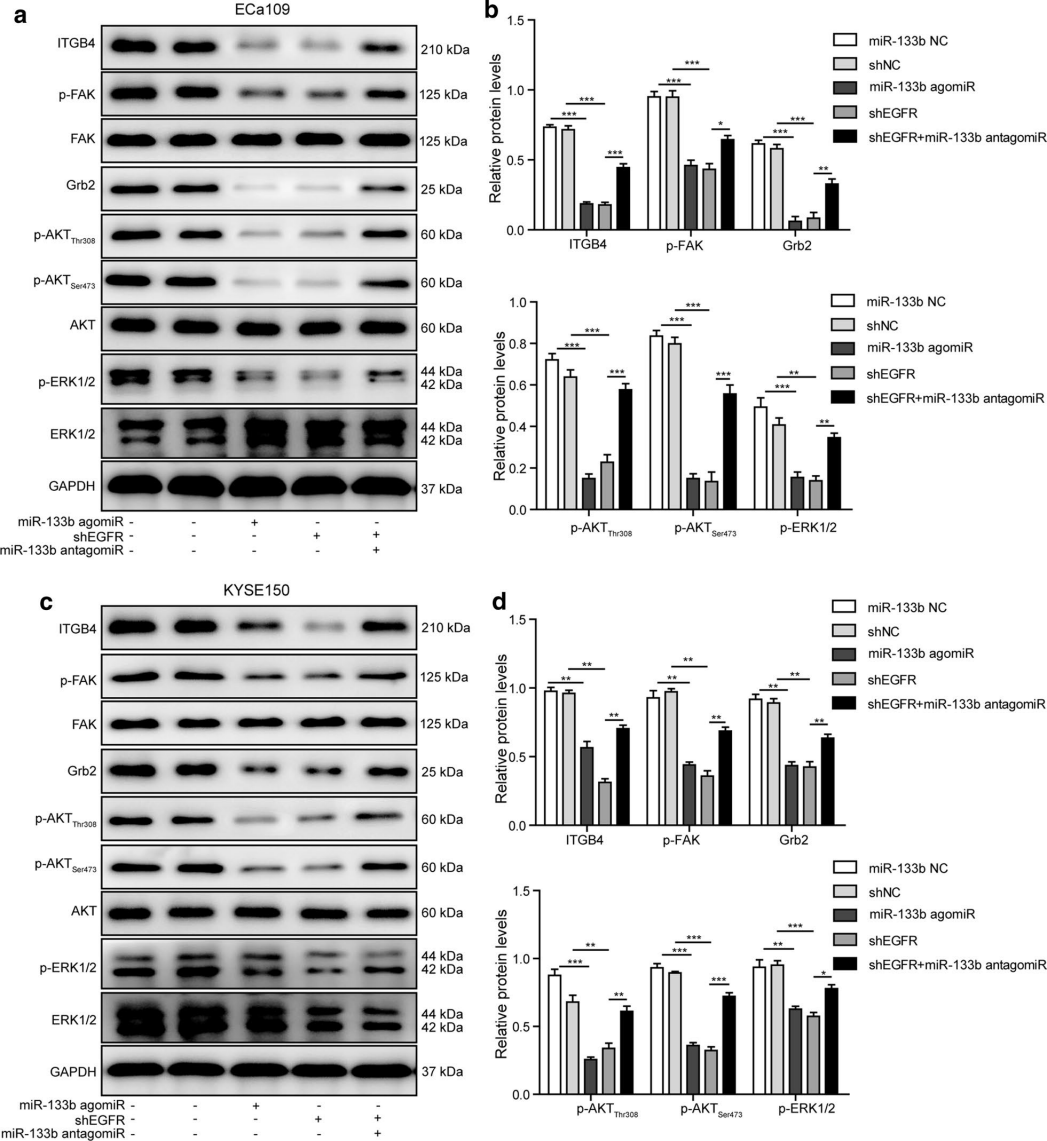
Regulation of miR-133b/EGFR axis in related signaling pathways of ESCC cells. **a**, **c** The protein levels of ITGB4, p-FAK, FAK, Grb2, p-AKT_Thr308_, p-AKT_Ser473_, p-ERK1/2, and ERK1/2 in KYSE150 and ECa109 cells were assessed by western blotting. **b**, **d** The gray-scale value of the bands were quantitatively analyzed. The experimental data were representative of three independent experiments. Results were expressed as mean ± SD. *P < 0.05, **P < 0.01 and ***P < 0.001

To elucidate the detailed mechanisms underlying the roles of miR-133b/EGFR axis in ESCC cells, a series of related molecules and signaling pathways were evaluated. As presented in Fig. 6a–d, miR-133b agomir or shEGFR caused obvious decrease in the protein levels of ITGB4, Grb2, p-FAK, p-AKT_Thr308_, p-ATK_Ser473_, and p-ERK1/2 in KYSE150 and ECa109 cells. But the reverse could occur, when the ESCC cells were co-transfected with miR-133b antagomir and shEGFR. Thus, ITGB4/FAK/Grb2, AKT and ERK pathways were involved in the mechanisms of miR-133b/EGFR axis in ESCC cells. MiR-133b/EGFR axis might regulate the proliferation, anoikis resistance and anchorage-independent growth, migration and invasion of ESCC cells through ITGB4/FAK/Grb2, AKT and ERK signaling pathways.

### MiR-133b inhibited tumor growth and lung metastasis of ESCC cells in mice

**Fig. 7:**
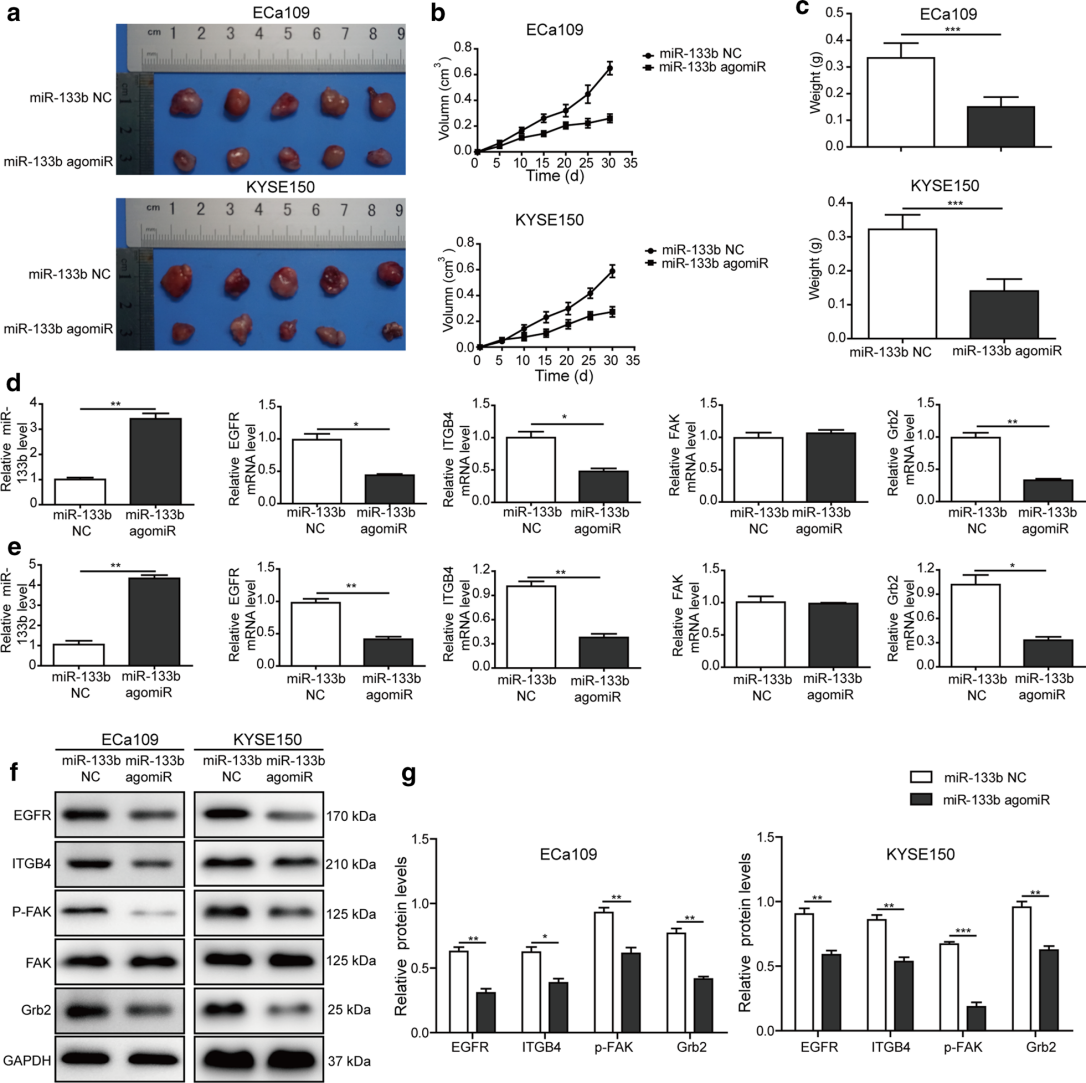
MiR-133b inhibited tumor growth and lung metastasis of ESCC cells in mice.(**a** The photograph of the xenograft tumors formed by KYSE150 and ECa109 cells transfected with miR-133b agomir or miR-133b NC at 30 days after injection. **b** Tumor growth curves and **c** tumor weight of each groups were shown. **d**, **e** The level of miR-133b, and mRNA levels of EGFR, ITGB4, FAK, and Grb2 in the lung tissues of mice injected with KYSE150 or ECa109 cells were detected by RT-qPCR. **f** The protein levels of EGFR, ITGB4, p-FAK, FAK, and Grb2 in the lung tissues were assessed by western blotting. **g** The gray-scale value of the bands were quantitatively analyzed. The experimental data were representative of three independent experiments. Results were expressed as mean ± SD. *P < 0.05, **P < 0.01 and ***P < 0.001

Finally, we further verified our results in a nude mice model in vivo. The results suggested that the tumor volume and weight were decreased in mice that were injected with miR-133b overexpressing KYSE150 and ECa109 cells (Fig. 7a–c). The level of miR-133b was raised, while EGFR, ITGB4, and Grb2 mRNA levels were reduced in the lung tissues of mice that were injected with miR-133b agomir-transfected KYSE150 and ECa109 cells (Fig. 7d, e). Concurrently, miR-133b agomir treatment led to the decreased protein levels of EGFR, ITGB4, p-FAK, and Grb2 in lung tissues of mice (Fig. 7f, g). The above results suggested that miR-133b inhibited the tumor growth and lung metastasis of ESCC cells in nude mice in vivo.

## Discussion

In this study, we aimed to evaluate the roles of miR-133b/EGFR axis in the metastasis of ESCC. Our results indicated that miR-133b level was down-regulated in the tissues and cells of ESCC, which promoted the proliferation, anoikis resistance, anchorage-independent growth and eventually led to ESCC invasion and metastasis via targeting EGFR and the downstream ITGB4/FAK/Grb2, AKT and ERK pathways. These results elucidated the mechanisms of invasion and metastasis of ESCC, which was a great help to bring more new strategies for ESCC therapy at the molecular level.

It was confirmed that miR-133b was down-regulated in multiple types of cancers, including ESCC, and played crucial roles in malignant progression of tumors. However, the detailed mechanisms of miR-133b in the regulation of invasion and metastasis of ESCC are not fully understood. Consistent with previous studies, our results indicated that the expression of miR-133b in ESCC tissues and cells was remarkably decreased. Moreover, miR-133b expression was negatively correlated with EGFR, ITGB4 and p-FAK levels in the tissues and cells of ESCC, suggesting these molecules may participate in the regulatory mechanisms of miR-133b in ESCC.

EGFR has been proved to be one of target genes of miR-133b in several human cancer cells [12, 13, 31]. It is well acknowledged that EGFR is a transmembrane glycoprotein with intracellular tyrosine kinase activity [32]. The activation of EGFR can trigger a series of downstream signaling pathways, principally the mitogen-activated protein kinase (MAPK) and PI3 K/AKT pathways [33] that are associated with proliferation, invasion and metastasis of cancer cells. Therefore, a number of anticancer drugs called “EGFR inhibitors” that targeting EGFR such as cetuximab and panitumumab have been adopted. In the present study, the results of dual luciferase reporter assay showed that EGFR was a target gene of miR-133b in ESCC cells, which further verified by the negative relationship between miR-133b and EGFR expression in ESCC tissues and cells. Moreover, overexpression of miR-133b restrained EGFR level in ESCC cells, whereas silencing of EGFR had no effect on miR-133b expression. These results provided evidence that in addition to EGFR, miR-133b might regulate the expression of other genes whose products were involved in EGFR signaling pathways.

Cancer cells detachment from ECM and the subsequent proliferation under anchorage-independent growth condition are considered as an early step of cancer metastasis [34]. Under normal condition, the detached normal cells may suffer apoptosis regulated by anoikis signaling pathways [35]. However, the tumor cells may survive and develop distant metastasis through anoikis resistance [36]. In this study, miR-133b overexpression and EGFR down-regulation suppressed anoikis resistance and anchorage-independent growth in ESCC cells via inhibiting EGFR expression. From these results, we speculated that miR-133b/EGFR might affect the migration and invasion of ESCC cells via regulating anoikis resistance and anchorage-independent growth. As expected, our results confirmed that the migration and invasion abilities of ESCC cells were repressed by miR-133b agomir and shEGFR via targeting EGFR. MMP-2 and MMP-9 are two important ECM degradation enzymes that promote tumor invasion and metastasis via breaking basement membrane structure and degrading ECM [37]. In this study, the protein levels of MMP-2 and MMP-9 in ESCC cells were suppressed by miR-133b overexpression and EGFR silencing. It is recognized that anoikis resistance facilitates metastasis via triggering EMT [18]. EMT is a process that well differentiated epithelial cells lose their polarity and cell–cell tight junctions, and transform to mesenchymal cells with increased motility and invasion abilities. Emerging data demonstrate that EMT is the driving force of cancer metastasis [38, 39]. During EMT process, the expression of E-cadherin, a epithelial marker, is decreased, while the expressions of mesenchymal markers, Fibronectin, Vimentin, and N-cadherin, are demonstrated to be increased [40]. According to the present study, the protein levels of Fibronection, Vimentin, and N-cadherin were down-regulated, while E-cadherin level was up-regulated in ESCC cells by miR-133b agomir or shEGFR treatment via targeting modulation of EGFR. Also, miR-133b antagomir reversed shEGFR-induced the changes in proliferation, anoikis resistance and anchorage-independent growth, migration and invasion of ESCC cells. From these results, we proposed that miR-133b/EGFR axis played pivotal roles in metastasis of ESCC via regulating anoikis resistance and anchorage-independent growth.

Furthermore, we focused on the detailed molecular mechanisms of miR-133b/EGFR axis in the regulation of anoikis resistance and metastasis of ESCC. Since we demonstrated that miR-133b expression was negatively correlated with ITGB4 and p-FAK levels in ESCC tissues and cells, the effects of miR-133b on ITGB4, FAK and related signaling pathways were further investigated. ITGB4 has been suggested to be up-regulated in multiple tumors and contribute to tumor progression by promoting proliferation, invasion and EMT process [41, 42]. A previous study showed that ITGB4 affected anoikis through interacting with EGFR in hepatocellular carcinoma [25]. ITGB4 was found to trigger the activation of downstream transmembrane protein kinases, including FAK, to protect against anoikis [43]. In addition, it was confirmed that FAK could bind to Grb2 and regulate the proliferation and invasion of melanoma [28]. There were a lot of downstream signaling pathways, such as AKT and ERK, that were regulated by Grb2 and facilitated tumor progression [30, 38]. In our study, miR-133b agomir or shEGFR inhibited the levels of ITGB4, Grb2, and the phosphorylation level of FAK, AKT and ERK. Our results indicated that miR-133b/EGFR axis regulated ITGB4/FAK/Grb2 pathway and downstream AKT and ERK pathways in ESCC cells, which might involve in the mechanisms of anoikis resistance and metastasis.

To further verify our findings, tumor xenografts in nude mice were performed. Consistent with the in vitro results, overexpression of miR-133b significantly restrained the tumor growth and lung metastases via regulating EGFR/ITGB4/FAK/Grb2 signaling pathway.

## Conclusions

Taken together, our data suggested that miR-133b was down-regulated in the tissues and cells of ESCC, which negatively correlated with EGFR, ITGB4, and p-FAK levels. Moreover, miR-133b inhibited the metastases of ESCC by regulating anoikis and anchorage-independent growth via targeting EGFR in vitro and in vivo. ITGB4/FAK/Grb2 and downstream AKT and ERK pathways participated in the regulatory mechanisms of miR-133b/EGFR axis in ESCC. Collectively, our results shed lights on the roles and mechanisms of miR-133b in ESCC metastases, which were involved in modulation of anoikis resistance and anchorage-independent growth. MiR-133b may be a potential novel diagnostic and therapeutic target against this malignancy.

## Abbreviations

ESCC: esophageal squamous cell carcinoma
miRNAs: microRNAs
UTRs: untranslated regions
EGFR: epidermal growth factor receptor
ECM: extracellular matrix
ITGB4: integrin β4
FAK: focal adhesion kinase
PI3K: phosphatidylinositol 3-kinase
AKT: protein kinase B
Grb2: growth factor receptor-bound protein 2
ERK: extracellular signal-regulated kinase
RPMI: Roswell Park Memorial Institute
FBS: fetal bovine serum
DMEM: Dulbecco’s Modified Eagle Medium
WT: wild-type
MUT: mutated
MTT: 3-(4,5-dimethylthiazol-2-yl)-2,5-diphenyltetrazolium bromide
DMSO: dimethyl sulfoxide
PBS: phosphate buffer solution
RT-qPCR: reverse transcription-quantitative polymerase chain reaction
GAPDH: glyceraldehyde-3-phosphate dehydrogenase
snRNA: small nuclear RNA
RIPA: radio immunoprecipitation assay
PMSF: phenylmethanesulfonyl fluoride
BCA: bicinchoninic acid
SDS-PAGE: sodium dodecyl sulphate-polyacrylamide gel electrophoresis
MMP-2: matrix metalloproteinase 2
SD: standard deviation
ANOVA: analysis of variance
EMT: epithelial-mesenchymal transition
MAPK: mitogen-activated protein kinase
